# Heart rate and cardiac response to exercise during voluntary dives in captive sea turtles (Cheloniidae)

**DOI:** 10.1242/bio.049247

**Published:** 2020-02-25

**Authors:** Junichi Okuyama, Maika Shiozawa, Daisuke Shiode

**Affiliations:** 1 Research Center for Subtropical Fisheries, Seikai National Fisheries Research Institute, Japan Fisheries Research and Education Agency, Ishigaki, Okinawa 907-0451, Japan; 2 Department of Marine Bioscience, Graduate School of Marine Science and Technology, Tokyo University of Marine Science and Technology, Minato, Tokyo 108-8477, Japan

**Keywords:** Bio-logging, Blood flow, *Chelonia mydas*, Electrocardiogram (ECG), Diving physiology

## Abstract

In chelonids, oxygen is primarily stored in the lungs during a dive. Therefore, management of blood oxygen transportation to peripheral tissues by cardiovascular adjustments during submergence is crucial to maximize their dive duration, and consequently, the time spent for ecological activities such as foraging. However, the cardiac response to exercise has rarely been examined in sea turtles. In this study, heart rate and its relationship with exercise during voluntary dives were determined in six captive green turtles (19.4±1.5 kg) by simultaneously recording depth, acceleration and electrocardiogram. Our results demonstrated that the heart rate of green turtles was generally low (11.1±0.4 bpm) during resting dives, but they often exhibited instantaneously extreme tachycardia (up to 78.4 bpm). Green turtles elevated their heart rate up to 39.8±1.5 bpm during ventilation after resting dives, while up to 33.1±1.4 bpm after active dives. The heart rate immediately elevated with onset of exercise, and increased linearly with exercise. This result may indicate that turtles immediately need to transport oxygen from the lungs to peripheral tissues by pulmonary and systemic circulations to meet the metabolic demands of exercise because they mainly store oxygen in their lungs.

## Abstract (precis)

**Summary:** Green turtles showed immediate cardiac response to exercise during submergence, presumably because they store oxygen primarily in the lungs and need blood circulation to meet the metabolic demand.

## INTRODUCTION

Many air-breathing vertebrates are known to exhibit bradycardia when submerged, which allows them to regulate their blood oxygen depletion rate, thereby conserving onboard oxygen stores (Kooyman, 1989; Butler and Jones, 1997). This physiological mechanism enables these animals to remain submerged for prolonged periods (Kooyman, 1989; Butler and Jones, 1997). However, a paradoxical situation is created because underwater activity, such as swimming, should promote an elevation in heart rate to support the increased demands of aerobic metabolism. Thus, the potential for conflict between diving bradycardia and exercise responses has been investigated for marine mammals (Fedak et al., 1988; Williams et al., 1999; Goldbogen et al., 2019), aquatic birds (Millard et al., 1973) and sea turtles (Butler et al., 1984; Southwood et al., 2003; Williams et al., 2019). These studies have found that air-breathing vertebrates elevated their heart rate during exercise even while being submerged. Recent bio-logging techniques, such as accelerometry, have enabled researchers to record fine-scale simultaneous measurements of quantitative activity level and heart rate in marine mammals, further demonstrating that during submergence the heart rate increases linearly with exercise intensity, but only when the exercise intensity is above a certain level (Davis and Williams, 2012; Williams et al., 2017). Unfortunately, such an instantaneous cardiac response during submergence has not yet been examined in many air-breathing divers except for a few species of marine mammals [Weddell seals, *Leptonychotes weddellii*; bottlenose dolphins, *Tursiops truncatus* (Davis and Williams, 2012); narwhals, *Monodon monoceros* (Williams et al., 2017)].

Sea turtles are ectothermic marine animals and have well-adjusted physiological functions for prolonged dives (reviewed by Lutcavage and Lutz, 1997; Williard, 2013). For instance, their considerably slow metabolism compared to that in marine mammals and aquatic birds enables turtles to undertake extremely prolonged dives (e.g. Hochscheid et al., 2005). While marine mammals mainly store oxygen in blood and tissues (Kooyman, 1989), in sea turtles, particularly in Cheloniidae species, the primary oxygen stores are the lungs (>70% of the oxygen present in whole body; Lutz and Bentley, 1985; Lutcavage and Lutz, 1997). Further, the myoglobin concentration of muscle in Cheloniidae species is similar to land mammals, but much less than that found in diving mammals and penguins (Lutz and Bentley, 1985). Therefore, the management of blood oxygen transportation to peripheral tissues (e.g. muscle) from the lungs by cardiovascular adjustments is crucial during submergence, in order to maximize dive duration and benefits of underwater activities, such as feeding, predator avoidance and mating. The cardiac responses of sea turtles to diving have been of particular interest to comparative biologists (Berkson, 1966; Davenport et al., 1982; Butler et al., 1984; West et al., 1992; Southwood et al., 1999, 2003; Williams et al., 2019). However, the majority of previous studies have been conducted under experimental conditions and in a limited space, and heart rate in free-ranging sea turtles has rarely been examined (but see Southwood et al., 1999). It is very difficult to conduct electrocardiography (ECG) in sea turtles owing to the limited space for electrode placement due to the shell, and also the background electrical disturbance generated from muscular contractions during locomotion (Southwood et al., 2003; Williams et al., 2019). Therefore, unlike for marine mammals, knowledge on cardiac responses and associated adjustments during submergence in sea turtles remains limited. In this context, the objectives of this study were to examine the general aspects of heart rate during voluntary dives in green sea turtles, *Chelonia mydas* (Linnaeus, 1758), within a captive setting, and to determine their cardiac responses to activity during submergence by simultaneous measurement of quantitative activity level and heart rate.

## RESULTS

### Extraction of heart rate

**Fig. 1.**
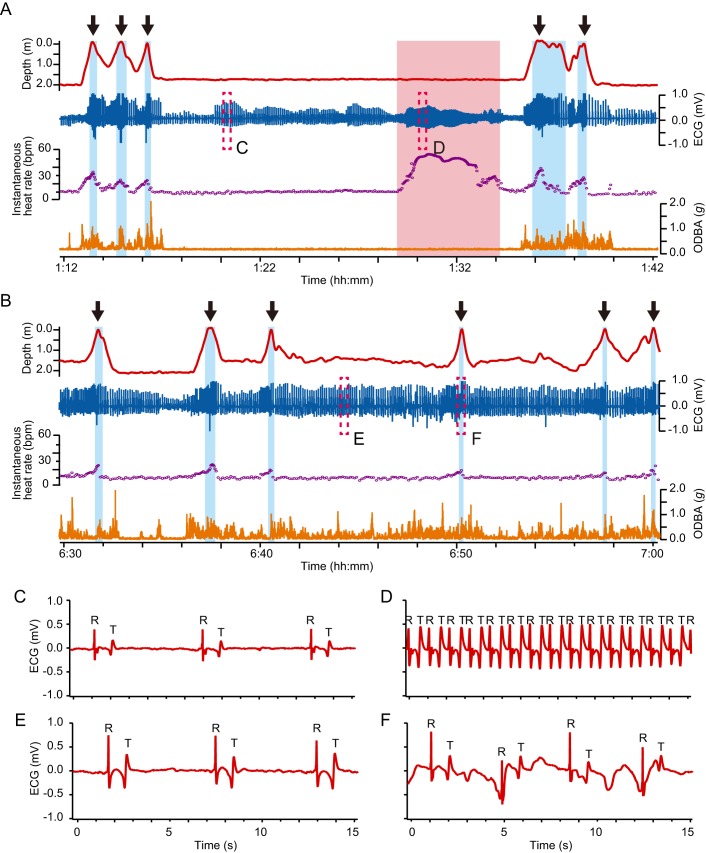
**Typical examples of ECG traces and cardiac responses during resting and active dives in a green turtle (CM5).** Typical profiles of depth, ECG, instantaneous heart rate and ODBA in (A) resting and (B) active dives. Blue and red backgrounds represent surface periods and extreme tachycardia periods, respectively. Black arrows represent breathing events. The ODBA values were calculated at each sampling interval of 3-axis acceleration (16 Hz or 20 Hz). Instantaneous heart rate was calculated from the intervals between consecutive R peaks. (C–F) Enlarged views of the ECG enclosed by dashed boxes in A and B. Typical ECG traces (C) in resting state, (D) during extreme tachycardia, (E) while swimming and (F) at surface ventilation. ‘R’ and ‘T’ labeled in these figures indicate the points of R peaks and T waves, respectively.

A total of 108.9 h of depth, temperature, 3-axis acceleration, and ECG data were obtained from six turtles (Table 1), of which 98.8 h (90.72%) worth of data free from any noise disturbance was used for analyses. Most of the ECG traces showed a clear QRS complex, but waveform and amplitude of the R peaks changed depending on the activity (Fig. 1; Figs S1–3). The amplitude of R peaks differed among individuals (Figs S1 and S2). P waves were not distinguishable in the majority of traces because of their small amplitude (Fig. S1). T waves were identified in ECG traces from all turtles and were recorded to be biphasic in our study (Fig. 1; Fig. S1).

**Table 1.**
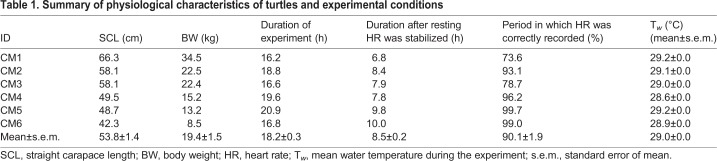
**Summary of physiological characteristics of turtles and experimental conditions**

**Fig. 2.**
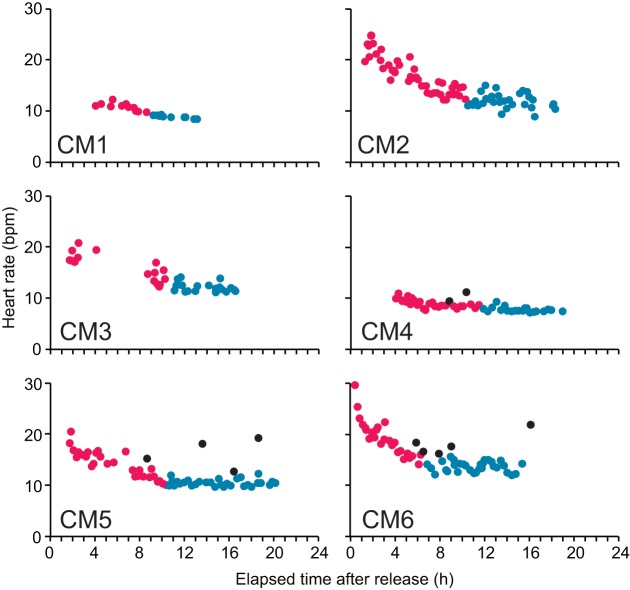
**Heart rates during the resting dives with elapsed time after release into the tank for each turtle.** Red and blue colors represent heart rate during the resting dives before and after it reached stable level, which was identified by visual observation. Black circles represent the heart rate during the dives including the extreme tachycardia phase. Sample sizes were 22, 75, 35, 50, 62 and 93 for CM1 to CM6, respectively.

The heart rate was observed to be higher during resting dives immediately after release, gradually decreasing and then stabilizing at a lower level within 6.8–11.8 h after release (Fig. 2, Table 1). As we restricted heart rate analyses to post-stabilization data, a total of 50.7 h of datasets, including heart rate, dive duration, mean ODBA and mean water temperature, available for 154 resting and 281 active dives were used (Tables 1, 2 and 3). Furthermore, we also used the data recorded, including heart rate during surface ventilation and number of breaths taken, available for surface periods following 118 resting and 240 active dives (Tables 2 and 3).

**Table 2.**
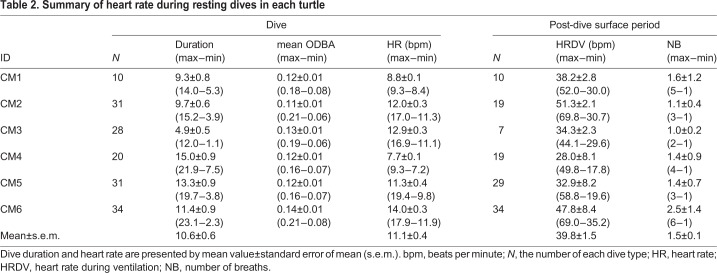
**Summary of heart rate during resting dives in each turtle**

**Table 3.**
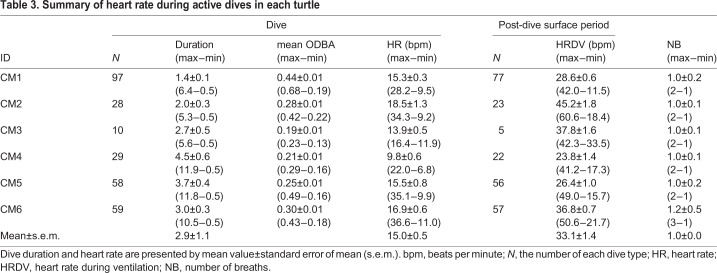
**Summary of heart rate during active dives in each turtle**

### Behavior of the turtles in the tank during the experiments

Turtles mostly performed active dives post release and were observed to be either swimming or crawling on the tank bottom during the day (6:30–19:00). Though resting dives were mostly performed during the night (19:00–6:30), they were also observed during the day. Erratic behavior was not detected on any occasion, neither from visual observations nor from depth and acceleration data. Mean dive duration for resting dives was 10.6±0.6 min [±standard error of the mean (s.e.m.)], while for active dives it was 2.9±1.1 min (Table 1). Mean ODBA during resting dives was 0.12±0.00 g, while during active dives was 0.28±0.01 g (Tables 2 and 3). Water temperature ranged from 28.3–30.3°C during the experiment (Tables 2 and 3). The average (±s.e.m.) lactate level in the venous blood immediately after the experiments was 1.0±0.2 mmol l^−^^1^.

### Heart rate during dives

**Fig. 3.**
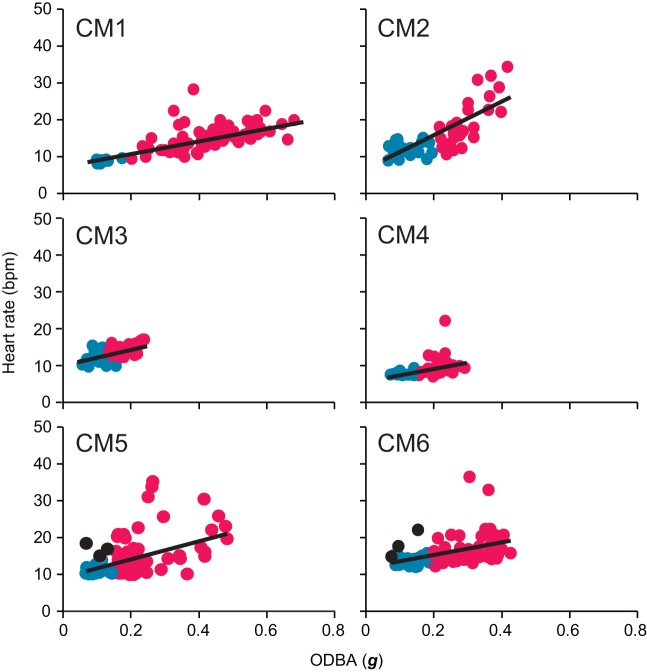
**Relationships between heart rate and mean ODBA during a dive in each turtle after heart rate had stabilized.** Blue and red circles represent the heart rate during resting and active dives, respectively. Black circles indicate dives including the extreme tachycardia phase. Sample sizes were 107, 59, 38, 49, 89 and 61 for CM1 to CM6, respectively. Black lines represent the linear regression line calculated using a least-square method.

The GLMM analysis showed that heart rate during a dive increased significantly with mean ODBA value (Fig. 3) and water temperature (Table 4), while it showed no significant relationship with individual body size (Table 4).

**Table 4.**
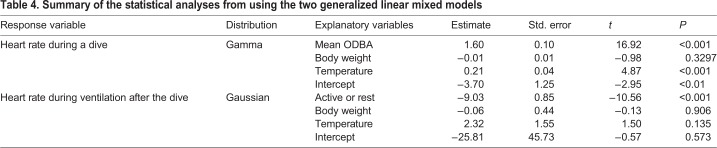
**Summary of the statistical analyses from using the two generalized linear mixed models**

Mean heart rate during resting dives was 11.1±0.4 bpm (±s.e.m.) (Table 2), while it was 15.0±0.5 bpm during active dives (Table 3). Instantaneous heart rate showed a quick response to an elevation in the activity levels (Fig. 1), increasing linearly with the mean ODBA (Fig. 3). It is noteworthy that in some cases, instantaneous heart rate (post-stabilization) showed instant and extreme tachycardia, even during resting dives (Fig. 1). Extreme tachycardia was defined as a heart rate that was more than twice the mean resting heart rate. We observed a total of 11 events of extreme tachycardia during the experiments, in individuals CM4, -5 and -6 (but only six events after the heart rate had stabilized from CM5 and -6), during which the maximum instantaneous heart rate ranged between 20.2 bpm and 78.4 bpm (Table 5). Such extreme tachycardia was observed mostly during the night (Table 5), and lasted for 133.0±8.5 s (Table 5).

**Table 5.**
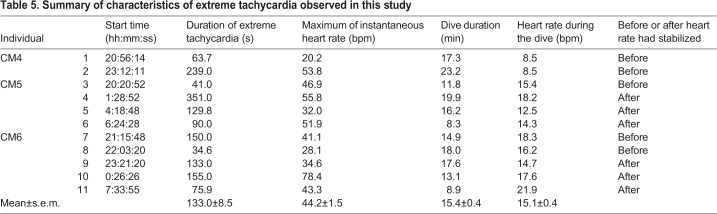
**Summary of characteristics of extreme tachycardia observed in this study**

### Cardiac responses to diving

The heart rate showed a clear reduction during submergence compared to surface period (Fig. 1). The instantaneous heart rate began to decrease immediately after the beginning of descent, while it increased concurrently with the start of ascent to the surface (Fig. 1). The time at maximum observed heart rate during the surface period coincided with the time of breathing (Fig. 1). Heart rate (±s.e.m.) during ventilation after resting dives was 38.4±1.5 bpm, exhibiting a 2.7–4.3-fold increase from heart rate during the dives (Table 2). However, heart rate during ventilation after active dives was 33.1±1.4 bpm, showing only a 1.7–2.7-fold increase from the heart rate observed during the dives (Table 3). Heart rate during ventilation after resting dives was significantly higher than after active dives (Table 4). Number of breaths during post-dive surface ventilation was 1.5±0.1 after resting dives and 1.0±0.0 after active dives (Tables 2 and 3).

## DISCUSSION

This study described the heart rate of green turtles during voluntary dives in captive conditions, and the changes therein caused by various factors, such as extent of activity and surface ventilation. Sea turtles are regarded as ‘surfacers’ rather than ‘divers’, because they spend most of their time underwater and surface only briefly for gas exchange (Kooyman, 1989). Therefore, a reduction in heart rate recorded during a dive in comparison to that at the surface is a normal condition for sea turtles.

### ECG recording in sea turtles

Several attempts have been made to obtain ECG recordings from wild as well as captive sea turtles, employing different patterns of electrode placement, i.e. placement in the region between the cervical regions and hindlimbs (Southwood et al., 1999; Harms et al., 2007; Williams et al., 2019) and on both sides of marginal scutes (Southwood et al., 2003). However, it has been difficult to record clear ECG traces from free-swimming sea turtles because of the disturbance from the electrical noise generated when turtles actively move (Southwood et al., 2003; Williams et al., 2019). In our study, we placed the two electrodes close together and over the heart to reduce noise, which allowed us to successfully record the heart rate during 90.1% of the experiment's duration, including swimming and surfacing periods.

Currently, the interpretation of the ECG for reptiles can be difficult because of the limited reference values available for comparison (Kik and Mitchell, 2005). The waveform and amplitude of ECG traces recorded in this study were difficult to compare with those from previous studies because of the differences in electrode placement. Furthermore, the waveform and amplitude changed depending on the activity even in an individual, presumably because the positions of the two electrodes relative to the heart changed slightly as a result of the turtles' movements. The difference in these ECG characteristics also reflected the difference in the electrode positions, relative to the heart, among the individuals. Most T waves recorded in this study were biphasic, similar to what was reported in nesting leatherback (Harms et al., 2007) and freshwater turtles (*Pseudemys elegans* and *Graptemys geographica*, Akers and Damm, 1963; *Mauremys japonica*, Zhang et al., 1987). Therefore, it is possible that some patterns of electrode placement – including those in our study – will produce biphasic traces of T waves. In the ECG trace recorded during extreme tachycardia, the amplitude of T wave became as large as the R peak, and the intervals between T wave and QRS complex were greatly reduced. Such compressed ECG traces were also recorded in freshwater turtles (*P. elegans* and *G. geographica*, Akers and Damm, 1963) when there was an extreme increase in the heart rate due to high core body temperature (>30°C). Thus, the compressed ECG trace seems to be observed when there is an extreme increase in the heart rate in sea turtles as well. These facts emphasize that extreme tachycardia did in fact occur during resting dives in our study.

### General perspective of heart rate in green turtles

Heart rate reduction after the animals were released in the tank took 6.8–11.8 h. This indicates that the turtles may have taken some time to reach their normal physiological rates after release, even though they were observed to have settled on the bottom of the tank. Heart rate is known to increase in response to handling stress in turtles (Cabanac and Bernieri, 2000) and in many other taxa (Tarlow and Blumstein, 2007), whereas it was reported to decrease under anesthesia in sea turtles (Chittick et al., 2002). Therefore, the stress caused by experimental handing, including the surgical attachment of electrodes (although the turtles were under anesthesia), and by release into a new environment (the experimental tank) from the rearing tank may have increased the heart rate. It took ∼6.8–11.8 h for the turtles to recover from this stress. This information should be taken into account by researchers when estimating metabolic rates of activity levels in sea turtles (e.g. Halsey et al., 2011) as the metabolic rate may be overestimated because of heart rate elevation resulting from a stress condition.

Most voluntary dives in sea turtles are assumed to be completed with aerobic metabolism (Butler et al., 1984; Okuyama et al., 2014). Despite the fact that turtles had to be chased to be captured, the concentration of lactate in venous blood collected immediately after the experiments (1.0±0.2 mmol l^−1^) was similar to that reported in previous studies (0.2–1.6 mmol l^−1^; Lutz and Bentley, 1985; Lutz et al., 1989), indicating that turtles mostly performed aerobic dives in our study. Our results demonstrated that in sea turtles, heart rate increased linearly with, and immediately following, an increase in the level of activity. However, simultaneous monitoring of ECG and exercise in marine mammals have shown that heart rate increases linearly with exercise (i.e. stroke frequency) during submergence, but only after the mammals have performed a certain amount of exercise during the dive (Davis and Williams, 2012; Williams et al., 2017). This time lag in heart rate elevation may reflect the differences in the storage of oxygen and thus blood circulation for oxygen delivery between marine mammals and sea turtles (Kooyman, 1989; Lutz and Bentley, 1985). Sea turtles need to transport oxygen from their lungs to peripheral tissues (e.g. muscle) by pulmonary and systemic circulations to meet the metabolic demand of exercise, while mammals can meet this metabolic demand by utilizing the oxygen already stored in peripheral tissues. These facts may indicate that sea turtles have a pronounced capability of efficiently transporting oxygen to active muscles from the lungs, depending on the metabolic demand during submergence. The extent of the increase in heart rate associated with activity (such as swimming) during a dive was not higher than that during surface ventilation and extreme tachycardia, indicating that usual activities, such as the swimming and crawling during submergence observed in this study, do not have excessive metabolic requirements, and consequently, do not require extensive cardiac adjustments. This further supports that turtles maintained aerobic metabolism during active dives.

Unlike mammalian and avian divers, the pulmonary and systemic circulations are not completely separated in reptiles including sea turtles, resulting in the possibility of central vascular shunts (Wang and Hicks, 1996). Thus, it is essential to understand how this intracardiac shunt occurs and how it changes in response to activity during submergence and surface ventilation, in order to determine how reptilian divers manage their blood oxygen transportation in pulmonary and systemic circulations. However, it was not possible to assess the presence of an intracardiac shunt from our ECG measurements. Previous laboratory studies investigating heart rate and pulmonary and arterial blood flow demonstrated that the net shunt was small during resting conditions in green turtles (Wood et al., 1984; West et al., 1992). Furthermore, during activity, pulmonary blood flow was always proportionately greater than arterial flow indicating that a net left-to-right shunts predominated (Wood et al., 1984; West et al., 1992). This also suggests that the right-to-left shunt is eliminated, or at least reduced during activity (Wang and Hicks, 1996). Because the change in pulmonary flow is synchronized with heart rate (Butler et al., 1984; West et al., 1992), the elevation in heart rate during active dives in our study presumably reflects the increase in pulmonary blood flow. These facts may indicate that green turtles facilitate oxygen uptake in the peripheral tissues by increasing the pulmonary circulation in relation to systemic circulation, in order to meet the oxygen demands of activity. This may represent a cardiovascular characteristic of chelonid divers that store their oxygen mainly in the lungs. However, further studies are required to investigate the relationship between heart rate and pulmonary and systemic circulations during voluntary dives of Cheloniid species.

Another factor affecting the heart rate is ambient water temperature (Davenport et al., 1982; Southwood et al., 2003). In our study, a significant effect of water temperature was observed on the heart rate during a dive (Table 4), although there was no substantial fluctuation in water temperature during the experiments (Table 1).

Previous laboratory studies reported that resting heart rates for juvenile green turtles ranged from 19.6–24 bpm during submerged in a small chamber or tank, with water temperatures similar to that in our study (26.0–28.8°C, Table 6). In terms of scaling, no significant effect of body size was detected in this study, probably because of the small sample size. Considering the data obtained from previous studies (Tables 1 and 6), the resting heart rate tended to decrease with increasing body weight. However, it is difficult to determine the effect of body size on heart rate from comparison with previous studies because of differences in measurement conditions and water temperature. Similar physiological parameters such as metabolic rate (Wallace and Jones, 2008) and aerobic dive limit (Hochscheid et al., 2007) were reported to have a relationship with body weight in sea turtles. The heart rates observed in our study seem to be lower in comparison with those of juvenile green turtles, with a similar body weight but recorded in slightly lower water temperatures (Table 6), reported in Southwood et al. (2003). This might be due to the difference in resting metabolic rates among populations (Kinoshita et al., 2018). Moreover, other factors such as holding conditions might play a role. Also, considering our turtles were wild while the turtles in Southwood et al. (2003) were captive-raised, the difference could have influenced the resting heart rate.

**Table 6.**
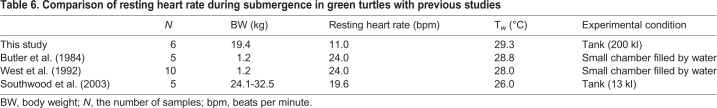
**Comparison of resting heart rate during submergence in green turtles with previous studies**

Heart rates of free-ranging leatherback turtles during diving during inter-nesting period in Costa Rica were documented as 17.4 bpm on average (Southwood et al., 1999), while their mean body temperature during inter-nesting intervals was reported to be around 30.2°C (Southwood et al., 2005). Thus, it can be expected to be similar for green turtles in this study, because body temperature of juvenile green turtles was reported to be 1.68°C above ambient water temperature at 30°C (Heath and McGinnis, 1980). In the study by Southwood et al. (1999), leatherbacks mostly conducted active dives. Considering the large size difference between adult leatherback turtles (250–400 kg; Southwood et al., 1999) and sub-adult green turtles (8.5–34.5 kg) in the present study, the heart rate in leatherbacks seems to be notably higher than that in green turtles during active dives. The allometric relationship between body mass and resting metabolic rate in leatherbacks is similar to that of green turtles (Wallace and Jones, 2008). However, leatherbacks show a higher active metabolic rate relative to their body size than do green turtles (Wallace and Jones, 2008), which might explain their higher heart rate during active dives. However, more data is required to determine the physiological reason for this difference in the heart rate between Cheloniidae and Dermochelyidae members.

### Extreme tachycardia during resting dives

Our study found that extreme tachycardia, i.e. an instantaneous and temporary elevation in heart rate, up to 78.4 bpm, often occurred during resting dives in green turtles. Irregular rhythms in heart rate or pulmonary blood flow have previously been reported in leatherbacks and green turtles (Davenport et al., 1982; West et al., 1992; Southwood et al., 1999) and in marine mammals (Williams et al., 2015). However, the extent of extreme tachycardia observed in our study greatly deviated from that of irregular cardiac rhythms. Such a phenomenon has never been reported in previous studies. Sanches et al. (2019) reported that, whilst mean heart rate in rattlesnake (*Crotalus durissus*) can quite rapidly (<24 h) return to resting levels following surgery and implantation of ECG electrodes, heart rate variability (respiratory sinus arrhythmia) takes much longer (i.e. 10 days) to fully recover. Therefore, the extreme tachycardia observed in our study is potentially caused by post-surgery stress, because our experiment recorded the heart rate within 24 h of the carrying out of surgical procedures for electrode attachment. Otherwise, extreme tachycardia might indicate that turtles temporarily increase blood flow to augment the oxygen supply to peripheral tissues (e.g. locomotory muscles), which may have experienced hypoxia, during a dive. It is unlikely to reflect a cardiac response resulting from a dominance of sympathetic activity due to stress or threat situation, because there was nothing in or around the tank that could possibly have threatened the turtles. Further research is required to determine if the observed extreme tachycardia is a natural physiological phenomenon or if it was in response to post-surgery stress.

### Cardiac response to surfacing

Many diving mammals and birds increase their heart rate towards the end of a dive in anticipation of surfacing (Butler and Jones, 1997), because an anticipatory tachycardia might restore blood flow to peripheral tissues, flushing out metabolites that may have built up and allowing for a more efficient removal of metabolic by-products and uptake of oxygen during recovery time at the surface (Thompson and Fedak, 1993). Our study demonstrated that green turtles also rapidly elevated their heart rate while ascending to the surface, as do free-ranging leatherbacks (Southwood et al., 1999) and captive Cheloniidae species (Butler et al., 1984; West et al., 1992; Williams et al., 2019).

Tachycardia during surface ventilation following resting dives was greater than that after active dives. Okuyama et al. (2014) suggested that green turtles almost completely deplete their oxygen stores during resting dives, replenishing them later at the surface by taking several breaths. Thus, higher tachycardia might indicate that green turtles adjust their cardiac response, in addition to increasing their breathing frequency, to facilitate rapid replenishment of oxygen and removal of CO_2_. As for active dives, green turtles do not completely deplete their oxygen stores; hence, such dives are followed by only a few breaths, which are sufficient for effective locomotion (Okuyama et al., 2014). Indeed, only a few breaths were observed during post-dive surface ventilation after active dives (Table 3), while several breaths were observed after resting dives in our study (Table 2). Thus, the partial pressure of remaining oxygen in the body after active dives may not aggravate tachycardia during post-dive ventilation.

In our study, green turtles exhibited a 3.5- and 2.2-fold increase in the heart rate during surface ventilation following resting and active dives, respectively (Tables 2 and 3). Based on the assumption that free-ranging dives are mostly active dives, the extent of tachycardia during surface ventilation in relation to the heart rate during a dive is not so pronounced in sea turtles [e.g. green turtles, 2.2-fold (this study), leatherback, 1.4-fold (Southwood et al., 1999); loggerheads, 2.4-fold (Williams et al., 2019)] when compared with marine mammals [e.g. harbor seals, *Phoca vitulina*, 3–4-fold (Fedak et al., 1988); northern elephant seals, *Mirounga angustirostris*, 2.8-fold (Andrews et al., 1997); and bottlenose dolphin, *Tursiops truncates*, 2.6-fold (Noren et al., 2012)]. This difference might be due to the fact that sea turtles need to transport oxygen from lungs to their muscles by blood circulation, particularly during active dives. As a result, the difference in the heart rate observed during active dives and surface ventilations is reduced. Conversely, marine mammals store their oxygen in muscle and blood tissues, and hence do not require as much blood circulation specifically from the lungs during a dive as do sea turtles. This enables them to maintain bradycardia during a dive (Kooyman, 1989), making the difference in the heart rate during dives and surface ventilations more pronounced.

### Conclusion

We successfully recorded clear ECG traces during submergence in Cheloniidae members under captive conditions, revealing their cardiovascular characteristics, particularly their cardiac response to exercise during submergence. Further comparison with marine mammals highlighted the characteristics of cardiac responses to exercise and ventilation in green turtles. These responses can be explained by a physiological characteristic of Cheloniidae species – the primary oxygen content is stored in their lungs, which consequently renders oxygen transportation from lungs to peripheral tissues crucial in order to meet the metabolic demands of exercise while submerged. We observed extreme tachycardia, where the heart rate instantaneously went up to 78.4 bpm, even during resting dives. Further studies are required to determine the reason behind such an unusual phenomenon. However, our findings would provide essential knowledge for understanding the diving physiology as well as the diving strategy in Cheloniidae species.

## MATERIALS AND METHODS

### Experimental protocol and instruments

All experiments were conducted in a tank (length×width×depth=10 m×10 m×2.2 m) at the Research Center for Subtropical Fisheries, Seikai National Fisheries Research Institute, Japan Fisheries Research and Education Agency (JFREA), on Ishigaki Island, Japan (24.46°N, 124.21°E), between August 21 and 30, 2018. Six wild green turtles were captured by a fisherman at the coastal reef habitat around Ishigaki Island under permits issued by the Okinawa Prefectural Government (No. K30-2) (Table 1). These turtles were captured 2 weeks before the experiments were conducted. They were maintained in two tanks (length×width×depth=8 m or 2.6 m×1.7 m×0.9 m) and were fed seagrass, brown algae (Sargassum sp.), and green algae (*Caulerpa lentillifera*) every day until the experiments.

**Fig. 4.**
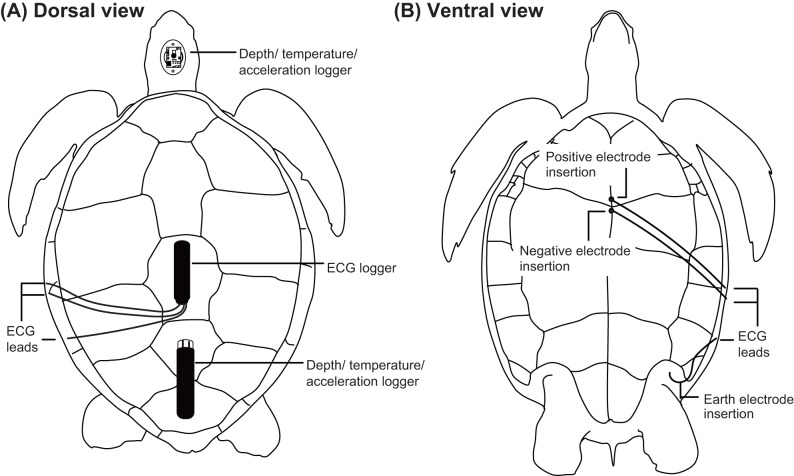
**Illustrations of data logger attachments on a green turtle.** (A) Dorsal view showing the placement of the data loggers and electrode leads on the carapace. (B) Ventral view showing the placement of three electrodes and leads on the plastron.

The experimental protocol and data logger attachments in this study were approved by Institutional Animal Care and Use Committees of both JFREA (permission No. 2018-001) and Tokyo University of Marine Science and Technology (permission No. H30-8). ECG traces of green turtles were recorded using an ECG data logger (W400-ECG, Little Leonard Co., Tokyo, Japan; 21 mm×109 mm cylindrical logger, 200 Hz sampling interval, voltage range±5.9 mV, 60 g, 2 GB memory). Three wires extending from the ECG logger were soldered to two stainless-steel electrodes (positive and negative, 0.9 mm in diameter, 30–35 mm in length) and a 21–23 gauge sterile needle that functioned as a ground connection. Lengths of the two electrodes were modified according to the size of each turtle to record clear heartbeats. The placement of these electrodes was conducted under anesthesia. Medetomidine (0.2 ml kg^−1^ of body weight, Fujita Pharmaceutical Co., Ltd., Tokyo, Japan) was used as a sedative and injected intramuscularly into the base of front flippers. Approximately 5 min after the sedative injection was given, anesthesia was initiated by slow injection of 1 ml diluted water with Propofol (0.01 ml kg^−1^; Mylan Inc., PA, USA) into the cervical vein. Two ECG electrodes, 8–10 mm apart, were fitted through a 1.5-mm hole in the plastron and placed over the heart (Fig. 4). Dental acrylic (Fuji IX GP; GC Co., Ltd., Tokyo, Japan) was used to anchor the electrodes and seal the hole. A ground (earth) electrode was inserted at the point where the skin fused with the plastron anterior to the hindlimb, such that it remained parallel to the plastron at a depth of 55 mm. The ground electrode was secured using a silylated urethane resin (Konishi Co., Ltd., Osaka, Japan). Electrode placement required less than 15 min. The ECG data logger was placed on the carapace using epoxy putty (Cemedine Co., Ltd., Tokyo, Japan). The three wires from the electrodes were then fixed on the carapace and plastron and covered using a silylated urethane resin (Konishi Co., Ltd., Osaka, Japan) and duct tape (Koyo Kagaku Co., Ltd., Tokyo, Japan) to prevent cable movement, which might cause electrical noise. After the attachment procedures for electrodes and loggers, atipamezole (0.3 ml kg^−1^ Mepatia; Fujita Pharmaceutical Co., Ltd.) was injected into the base of front flippers as a medetomidine antagonist, to allow the turtle to recover from sedation. Then, each turtle was maintained in a plastic container (length×width×depth=1 m×0.7 m×0.2 m) for ∼1–1.5 h before the epoxy putty and resins completely hardened.

Heart rate, depth, temperature and 3-axis acceleration were simultaneously recorded using a multi-channel data logger placed on the carapace using epoxy putty (Fig. 4). We used either of the two types of loggers: ORI400-D3GT (12 mm diameter, 45 mm length, 9 g in air, memory capacity of 11.5 million data points, sampling rate: depth and temperature 1 Hz, acceleration 20 Hz, maximum range of depth sensor 400 m with a resolution of 0.1 m, measurement range of accelerometers ±3 ***g*** with a resolution of 0.00009 ***g***, Little Leonardo Co.) or W380-PD3GT (21 mm diameter, 114 mm length, 59 g in air, memory capacity 16 million data points, sampling rate: depth and temperature 1 Hz, acceleration 16 Hz, maximum range of depth sensor 380 m with a resolution of 0.1 m, measurement range of accelerometers ±4 ***g*** with a resolution of 0.002 ***g***, Little Leonardo Co.). Furthermore, the number of breaths taken at the surface after each dive was counted by the head-mounted acceleration data logger (G6a+; dimensions: 40 mm×28 mm×17 mm, weight: 19 g in air; measurement range: ±2 ***g*** with a resolution of 0.002 ***g***, Cefas Technology Limited, Suffolk, UK; Fig. 4).

Acceleration data were separated into two components: the high frequency component, which was a dynamic acceleration representing turtle movement (such as swimming), and the low frequency component, which was gravity acceleration representing the turtle's body angle (Okuyama et al., 2012). This frequency separation was conducted by filtering analysis [see Okuyama et al. (2012) for details]. Moreover, acceleration data were used to calculate the amount of activity performed by each turtle, which was given by overall dynamic body acceleration (ODBA; Wilson et al., 2006). ODBA was used as a proxy of the amount of activity in sea turtles (Halsey et al., 2011; Okuyama et al., 2014). ODBA was calculated from the high frequency component of the 3-axis acceleration data. Head angle of sea turtles, calculated from the low frequency component of acceleration data, and depth data allowed interpretation of their breathing activity (Okuyama et al., 2010). Briefly, turtles were considered to be breathing when they raised their head up at an angle >30° at a depth <0.15 m (Okuyama et al., 2010).

Each turtle equipped with the data loggers was then released into the experiment tank and allowed to swim freely for 16.3–21.5 h (from the afternoon to the next morning). Temperature during the experiments ranged from 28.6–29.2°C (Table 1). Turtles were not fed during experiments. After the experiments, turtles were immediately removed from the tank. Blood samples were collected from the cervical vein using a syringe (1 ml, Nipro Co., Osaka, Japan) and a needle (20-gauge, Terumo Co., Tokyo, Japan) within a minute of capture. Lactate levels in venous blood was measured using an i-STAT 1 Analyzer (Abbott Point of Care Inc., IL, USA) to examine the possibility that turtles engaged in anaerobic dives during experiments. To the best of our knowledge, the measurement of lactate levels for sea turtles by i-STAT provides a reliable value without temperature correction (Harms et al., 2003; Muñoz-Pérez et al., 2016; Yang et al., 2019). After blood sampling, all electrodes were removed, and the holes were filled with dental acrylic. The turtles were maintained in the rearing tank while the holes healed, and were released back into the sea 2 weeks after the experiments, when the holes had completely closed.

### Extraction of heart rate from ECG data and data analysis

Data of ECG, depth and acceleration were analyzed using Igor Pro version 6.36 (Wavemetrics, OR, USA). Although P and T waves could not be always confirmed in all ECG traces, the R peak in the QRS complex (the main graphical deflections in an ECG tracing; Fig. 1C–F; Fig. S1) was detectable in most cases, and thus, was regarded as one heartbeat. However, when the heart rate was higher, the amplitude of the positive peak of T wave became as large as the R peak (Fig. 1D; Fig. S2). In such cases, we discriminated the R peaks from the positive peaks of T waves by the fact that clear T waves always exhibited a biphasic waveform where a negative peak was immediately followed by a positive one (Fig. 1C–F; Figs S1 and S2), while no clear downward deflection of Q waves in the QRS complex was observed.

The intervals between two consecutive R peaks (R-R intervals) were used to calculate the instantaneous heart rate for the analysis of instantaneous cardiac response (Williams et al., 2019). Additionally, the number of R peaks was counted for each dive. A dive was defined as the period when turtles submerged deeper than 0.5 m for longer than 30 s; the remaining period was regarded as surface period. Thus, the average heart rate during a dive was calculated by dividing the number of R peaks during the dive by the dive duration. The instantaneous heart rate recorded during or immediately before breathing event at the post-dive surface period was considered the heart rate during surface ventilation, because the breathing activity derived from acceleration and depth data generally occurred over only less than 2 s (Fig. 1, Okuyama et al., 2010) and, therefore, instantaneous heart rate was not always recorded during breathing events. Mean value of ODBA during a dive was also calculated as an index of the amount of activity. The number of breathing events occurring during the surface period was counted. Mean water temperature was calculated during each dive, and it was used to investigate the relationship between heart rate and temperature for each dive.

In some cases, disturbance from background noise did not allow the identification of R peaks (see the Results section; Fig. S3). If such noise data was recorded during the dive period, the calculations of heart rate and mean ODBA for that dive were made based on the dive duration remaining after the elimination of the dive segment with noise. However, if the noise was recorded during the surface period, it had to be completely excluded from further analysis, because the surface period remaining after eliminating the segment with noise was too short to calculate heart rate.

To investigate resting heart rate, and also to understand the relationship between heart rate and activity levels, the dives were categorized into two types: resting and active dives. A resting dive was defined as a dive when the turtles remained still at the bottom of tank, except for descending and ascending phases. Still condition was characterized by constant dynamic acceleration, body angle and depth (2.0 m) (Fig. 1A). Percentage of still condition during resting dives ranged from 75–95% (see Fig. 1A), but it varied with dive duration. All other dives were defined as active dives.

Because the heart rate during resting dives appeared to decrease with time elapsed after the release, before stabilizing (see the Results section; Fig. 2), the time of reaching this stable level was visually identified based on Fig. 2. Thus, the heart rate at the stable level was regarded as the normal resting heart rate for each turtle. To correctly examine the heart rates, we restricted the heart rate analyses to data obtained after the heart rate had stabilized.

### Statistical analysis

A generalized linear mixed model (GLMM) with a Gamma distribution and a log link function was used to determine the factors affecting mean heart rate during a dive. Mean ODBA during a dive, body weight of turtles and mean water temperature during a dive were treated as explanatory variables (Table 1). Individual was treated as a random effect. We also investigated the factors affecting mean heart rate during the post-dive surface period using a GLMM with a Gaussian distribution and a log link function. Activity during a dive was converted to dummy (binary) variables (resting dives=0, active dives=1) and treated as an explanatory variable. Moreover, body weight and water temperature were also treated as explanatory variables and individual was treated as a random effect. The ‘lme4’ package in R v. 3.5.2 (R Development Core Team, 2018) software was used to run GLMM analyses.

## Supplementary Material

Supplementary information

## References

[BIO049247C1] Akers T. K. and Damm M. G. (1963). The effect of temperature on the electrocardiograms of two species of turtles. *Copeia* 1963, 629-634. 10.2307/1440963

[BIO049247C2] Andrews R. D., Jones D. R., Williams J. D., Thorson P. H., Oliver G. W., Costa D. P. and Le Boeuf B. J. (1997). Heart rates of northern elephant seals diving at sea and resting on the beach. *J. Exp. Biol.* 200, 2083-2095. 9255950 10.1242/jeb.200.15.2083

[BIO049247C3] Berkson H. (1966). Physiological adjustments to prolonged diving in the Pacific green turtle (*Chelonia mydas agassizii*). *Comp. Biochem. Physiol.* 18, 101-119. 10.1016/0010-406X(66)90335-5 5965104

[BIO049247C4] Butler P. J. and Jones D. R. (1997). Physiology of diving of birds and mammals. *Physiol. Rev.* 77, 837-899. 10.1152/physrev.1997.77.3.837 9234967

[BIO049247C5] Butler P. J., Milsom W. K. and Woakes A. J. (1984). Respiratory, cardiovascular and metabolic adjustments during steady state swimming in the green turtle, *Chelonia mydas*. *J. Comp. Physiol. B* 154, 167-174. 10.1007/BF00684141

[BIO049247C6] Cabanac M. and Bernieri C. (2000). Behavioral rise in body temperature and tachycardia by handling of a turtle (*Clemmys insculpta*). *Behav. Processes* 49, 61-68. 10.1016/S0376-6357(00)00067-X 10794915

[BIO049247C7] Chittick E. J., Stamper M. A., Beasley J. F., Lewbart G. A. and Horne W. A. (2002). Medetomidine, ketamine, and sevoflurane for anesthesia of injured loggerhead sea turtles. *J. Am. Vet. Med. Assoc.* 221, 1019-1025. 10.2460/javma.2002.221.1019 12369681

[BIO049247C8] Davenport J., Inagle G. and Hughes A. K. (1982). Oxygen uptake and heart rate in young green turtles (*Chelonia mydas*). *J. Zool.* 198, 399-412. 10.1111/j.1469-7998.1982.tb02084.x

[BIO049247C9] Davis R. W. and Williams T. M. (2012). The marine mammal dive response is exercise modulated to maximize aerobic dive duration. *J. Comp. Physiol. A* 198, 583-591. 10.1007/s00359-012-0731-4 22585422

[BIO049247C10] Fedak M. A., Pullen M. R. and Kanwisher J. (1988). Circulatory responses of seals to periodic breathing: heart rate and breathing during exercise and diving in the laboratory and open sea. *Can. J. Zool.* 66, 53-60. 10.1139/z88-007

[BIO049247C11] Goldbogen J. A., Cade D. E., Calambokidis J., Czapanskiy M. F., Fahlbusch J., Friedlaender A. S., Gough W. T., Kahane-Rapport S. R., Savoca M. S., Ponganis K. V. et al. (2019). Extreme bradycardia and tachycardia in the world's largest animal. *Proc. Natl. Acad. Sci. USA* 116, 25329-25332. 10.1073/pnas.1914273116 31767746 PMC6911174

[BIO049247C12] Halsey L. G., Jones T. T., Jones D. R., Liebsch N. and Booth D. T. (2011). Measuring energy expenditure in sub-adult and hatchling sea turtles via accelerometry. *PLoS ONE* 6, e22311 10.1371/journal.pone.0022311 21829613 PMC3150346

[BIO049247C13] Harms C. A., Mallo K. M., Ross P. M. and Segars A. (2003). Venous blood gases and lactates of wild loggerhead sea turtles (*Caretta caretta*) following two capture techniques. *J. Wildl. Dis.* 39, 366-374. 10.7589/0090-3558-39.2.366 12918445

[BIO049247C14] Harms C. A., Eckert S. A., Kubis S. A., Campbell M., Levenson D. H. and Crognale M. A. (2007). Field anaesthesia of leatherback sea turtles (*Dermochelys coriacea*). *Vet. Rec.* 161, 15-21. 10.1136/vr.161.1.15 17617540

[BIO049247C15] Heath M. E. and McGinnis S. M. (1980). Body temperature and heat transfer in the green sea turtle, *Chelonia mydas*. *Copeia* 1980, 767-773. 10.2307/1444455

[BIO049247C16] Hochscheid S., Bentivegna F. and Hays G. C. (2005). First records of dive durations for a hibernating sea turtle. *Biol. Lett.* 1, 82-86. 10.1098/rsbl.2004.0250 17148134 PMC1629053

[BIO049247C17] Hochscheid S., McMahon C. R., Bradshaw C. J. A., Maffucci F., Bentivegna F. and Hays G. C. (2007). Allometric scaling of lung volume and its consequences for marine turtle diving performance. *Comp. Biochem. Physiol. A* 148, 360-367. 10.1016/j.cbpa.2007.05.010 17596981

[BIO049247C18] Kik M. J. L. and Mitchell M. A. (2005). Reptile cardiology: a review of anatomy and physiology, diagnostic approaches, and clinical disease. *Semin. Avian Exot. Pet Med.* 14, 52-60. 10.1053/j.saep.2005.12.009

[BIO049247C19] Kinoshita C., Fukuoka T., Niizuma Y., Narazaki T. and Sato K. (2018). High resting metabolic rates with low thermal dependence induce active dives in overwintering Pacific juvenile loggerhead turtles. *J. Exp. Biol.* 221, jeb175836 10.1242/jeb.175836 29748215

[BIO049247C20] Kooyman G. L. (1989). *Diverse Divers*. Berlin, Germany: Springer.

[BIO049247C21] Lutcavage M. E. and Lutz P. L. (1997). Diving physiology. In *The Biology of Sea Turtles* (ed. Lutz P. L. and Musick J. A.), pp. 277-296. Boca Raton, FL: CRC Press.

[BIO049247C22] Lutz P. L. and Bentley T. B. (1985). Respiratory physiology of diving in the sea turtle. *Copeia* 1985, 671-679. 10.2307/1444761

[BIO049247C23] Lutz P. L., Bergey A. N. N. and Bergey M. (1989). Effects of temperature on gas exchange and acid-base balance in the sea turtle *Caretta caretta* at rest and during routine activity. *J. Exp. Biol.* 144, 155-169.

[BIO049247C24] Millard R. W., Johansen K. and Milsom W. K. (1973). Radiotelemetry of cardiovascular responses to exercise and diving in penguins. *Comp Biochem Physiol A* 46, 227-240. 10.1016/0300-9629(73)90414-3 4147892

[BIO049247C25] Muñoz-Pérez J. P., Lewbart G. A., Hirschfeld M., Alarcón-Ruales D., Denkinger J., Castañeda J. G., García J. and Lohmann K. J. (2017). Blood gases, biochemistry and haematology of Galápagos hawksbill turtles (*Eretmochelys imbricata*). *Conserv. Physiol.* 5, cox028 10.1093/conphys/cox028 28496982 PMC5424066

[BIO049247C26] Noren S. R., Kendall T., Cuccurullo V. and Williams T. M. (2012). The dive response redefined: underwater behavior influences cardiac variability in freely diving dolphins. *J. Exp. Biol.* 215, 2735-2741. 10.1242/jeb.069583 22837445

[BIO049247C27] Okuyama J., Kawabata Y., Naito Y., Arai N. and Kobayashi M. (2010). Monitoring beak movements with an acceleration datalogger: a useful technique for assessing the feeding and breathing behaviors of sea turtles. *Endang. Species. Res.* 10, 39-45. 10.3354/esr00215

[BIO049247C28] Okuyama J., Kataoka K., Kobayashi M., Abe O., Yoseda K. and Arai N. (2012). The regularity of dive performance in sea turtles: a new perspective from precise activity data. *Anim. Behav.* 84, 349-359. 10.1016/j.anbehav.2012.04.033

[BIO049247C29] Okuyama J., Tabata R., Nakajima K., Arai N., Kobayashi M. and Kagawa S. (2014). Surfacers change their dive tactics depending on the aim of the dive: evidence from simultaneous measurements of breaths and energy expenditure. *Proc. R. Soc. B* 281, 20140040 10.1098/rspb.2014.0040 PMC4213604 25297856

[BIO049247C30] R Development Core Team (2013). *R: A Language and Environment for Statistical Computing*. Vienna, Austria: R Foundation for Statistical Computing.

[BIO049247C31] Sanches P. V. W., Taylor E. W., Duran L. M., Cruz A. L., Dias D. P. M. and Leite C. A. C. (2019). Respiratory sinus arrhythmia is a major component of heart rate variability in undisturbed, remotely monitored rattlesnakes, *Crotalus durissus*. *J. Exp. Biol.* 222, jeb197954 10.1242/jeb.197954 30967516

[BIO049247C32] Southwood A. L., Andrews R. D., Lutcavage M. E., Paladino F. V., West N. H., George R. H. and Jones D. R. (1999). Heart rates and diving behavior of leatherback sea turtles in the eastern Pacific Ocean. *J. Exp. Biol.* 202, 1115-1125. 10101109 10.1242/jeb.202.9.1115

[BIO049247C33] Southwood A. L., Darveau C. A. and Jones D. R. (2003). Metabolic and cardiovascular adjustments of juvenile green turtles to seasonal changes in temperature and photoperiod. *J. Exp. Biol.* 206, 4521-4531. 10.1242/jeb.00689 14610036

[BIO049247C34] Southwood A. L., Andrews R. D., Paladino F. V. and Jones D. R. (2005). Effects of diving and swimming behavior on body temperatures of Pacific leatherback turtles in tropical seas. *Physiol. Biochem. Zool.* 78, 285-297. 10.1086/427048 15778947

[BIO049247C35] Tarlow E. M. and Blumstein D. T. (2007). Evaluating methods to quantify anthropogenic stressors on wild animals. *Appl. Anim. Behav. Sci.* 102, 429-451. 10.1016/j.applanim.2006.05.040

[BIO049247C36] Thompson D. and Fedak M. A. (1993). Cardiac responses of grey seals during diving at sea. *J. Exp. Biol.* 174, 139-164. 8440964 10.1242/jeb.174.1.139

[BIO049247C37] Wallace B. P. and Jones T. T. (2008). What makes marine turtles go: a review of metabolic rates and their consequences. *J. Exp. Mar. Biol. Ecol.* 356, 8-24. 10.1016/j.jembe.2007.12.023

[BIO049247C38] Wang T. and Hicks J. W. (1996). The interaction of pulmonary ventilation and the right-left shunt on arterial oxygen levels. *J. Exp. Biol.* 199, 2121-2129. 8896361 10.1242/jeb.199.10.2121

[BIO049247C39] West N. H., Butler P. J. and Bevan R. M. (1992). Pulmonary blood flow at rest and during swimming in the green turtle, *Chelonia mydas*. *Physiol. Zool.* 65, 287-310. 10.1086/physzool.65.2.30158254

[BIO049247C40] Williams T. M., Haun J. E. and Friedl W. A. (1999). The diving physiology of bottlenose dolphins (*Tursiops truncatus*). I. Balancing the demands of exercise for energy conservation at depth. *J. Exp. Biol.* 202, 2739-2748. 10504310 10.1242/jeb.202.20.2739

[BIO049247C41] Williams T. M., Fuiman L. A., Kendall T., Berry P., Richter B., Noren S. R., Thometz N., Shattock M. J., Farrell E., Stamper A. M. et al. (2015). Exercise at depth alters bradycardia and incidence of cardiac anomalies in deep-diving marine mammals. *Nat. Commun.* 6, 6055 10.1038/ncomms7055 25592286

[BIO049247C42] Williams T. M., Blackwell S. B., Richter B., Sinding M. H. S. and Heide-Jørgensen M. P. (2017). Paradoxical escape responses by narwhals (*Monodon monoceros*). *Science* 358, 1328-1331. 10.1126/science.aao2740 29217577

[BIO049247C43] Williams C. L., Sato K. and Ponganis P. J. (2019). Activity, not submergence, explains diving heart rates of captive loggerhead sea turtles. *J. Exp. Biol.* 222, jeb200824 10.1242/jeb.200824 30936271

[BIO049247C44] Williard A. S. (2013). Physiology as integrated systems. In *The Biology of Sea Turtles*, Vol. III (ed. Wyneken J., Lohmann K. J. and Musick J. A.), pp. 1-30. Boca Raton, FL: CRC Press.

[BIO049247C45] Wilson R. P., White C. R., Quintana F., Halsey L. G., Liebsch N., Martin G. R. and Butler P. J. (2006). Moving towards acceleration for estimates of activity-specific metabolic rate in free-living animals: the case of the cormorant. *J. Anim. Ecol.* 75, 1081-1090. 10.1111/j.1365-2656.2006.01127.x 16922843

[BIO049247C46] Wood S. C., Gatz R. N. and Glass M. L. (1984). Oxygen transport in the green sea turtle. *J. Comp. Physiol. B* 154, 275-280. 10.1007/BF02464407

[BIO049247C47] Yang T., Haas H. L., Patel S., Smolowitz R., James M. C. and Williard A. S. (2018). Blood biochemistry and haematology of migrating loggerhead turtles (*Caretta caretta*) in the Northwest Atlantic: reference intervals and intra-population comparisons. *Conserv. Physiol.* 7, coy079 10.1093/conphys/coy079 PMC6366141 30746149

[BIO049247C48] Zhang K. X., Ohno K. and Kadono H. (1987). Studies on the electrocardiogram of the tortoise: lead methods and standard values. *Res. Bull. Fac. Agr. Gifu. Univ.* 52, 191-198. (in Japanese with English abstract).

