# Supplementary material for: Heart rate and cardiac response to exercise during voluntary dives in captive sea turtles (Cheloniidae)
Source: Biol Open. 2020 Feb 25;9(2):bio049247. doi: 10.1242/bio.049247 (PMC7055368; doi:10.1242/bio.049247)
Supplement: Supplementary information [file biolopen-9-049247-s1.pdf]

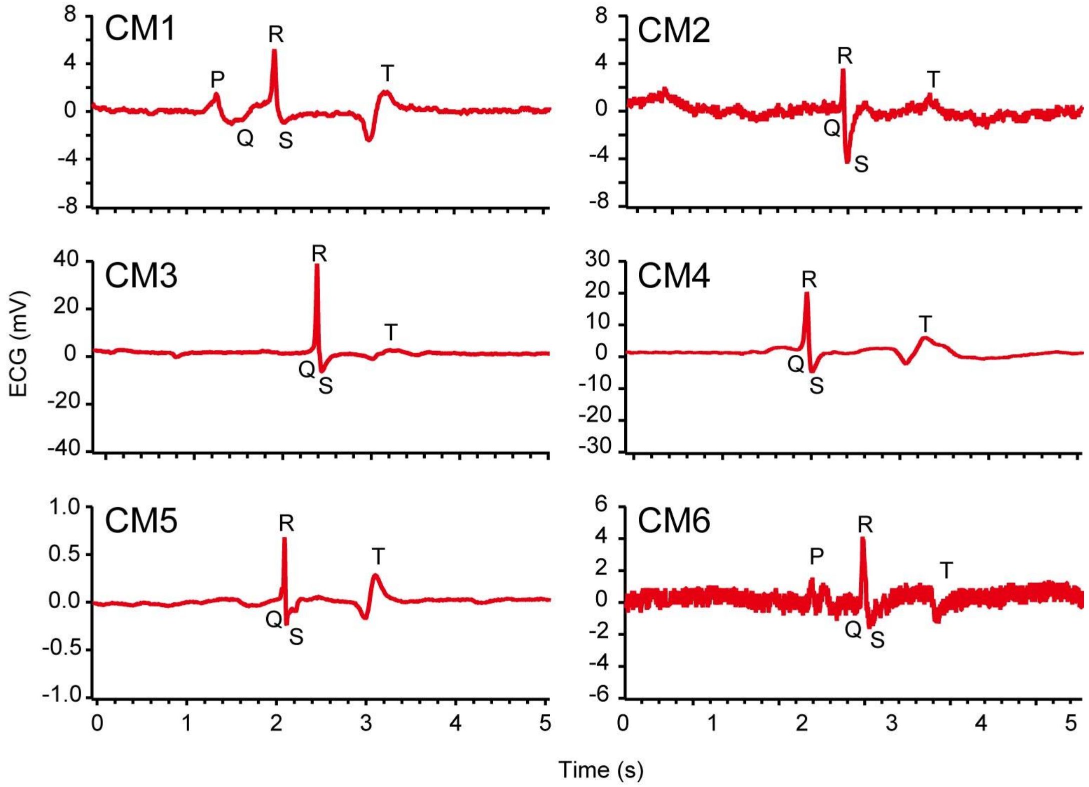

Fig. S1. Typical ECG traces in each turtle. ‘P’, ‘Q’, ‘R’, ‘S’ and ‘T’ labeled in the figures indicate the points of P-, Q-, R-, S-, T-waves, respectively. In CM2 to CM5, we did not identify the P-wave from the ECG trace.

Biology Open • Supplementary information

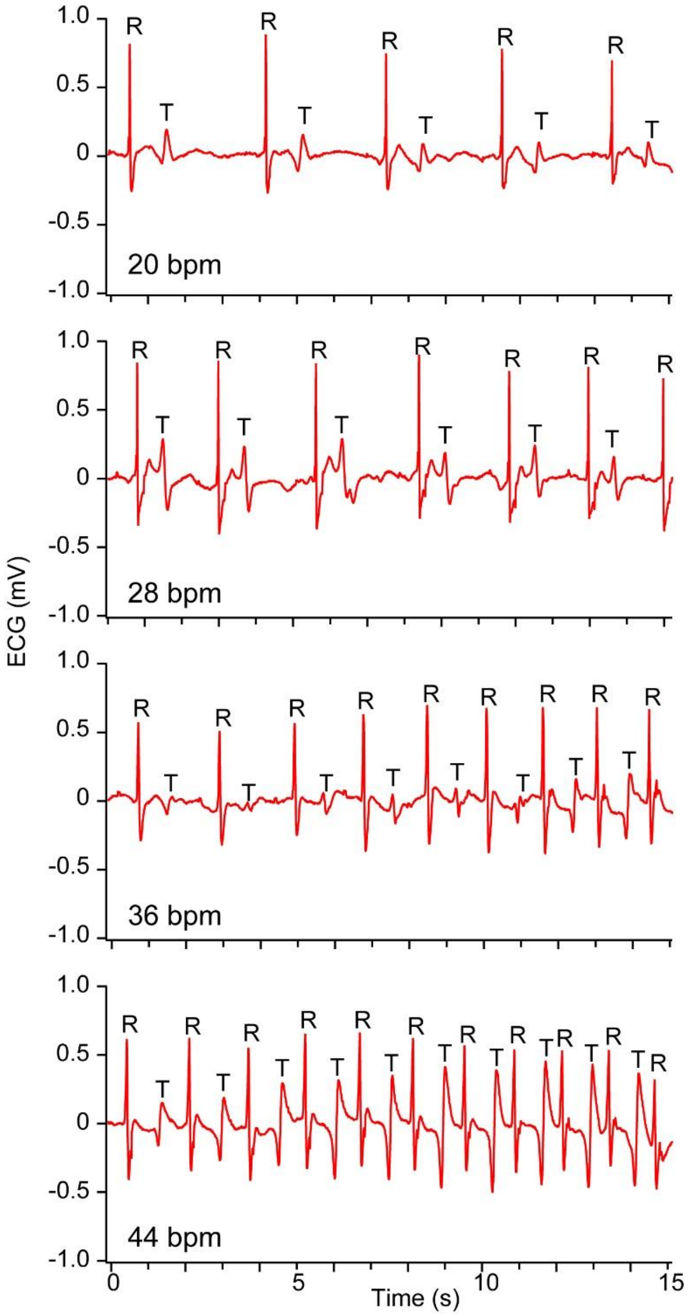

Fig. S2. Typical ECG traces at various heart rates in CM5. 'R' and 'T' labeled in the figures indicate the points of R peak and T wave, respectively.

Biology Open • Supplementary information

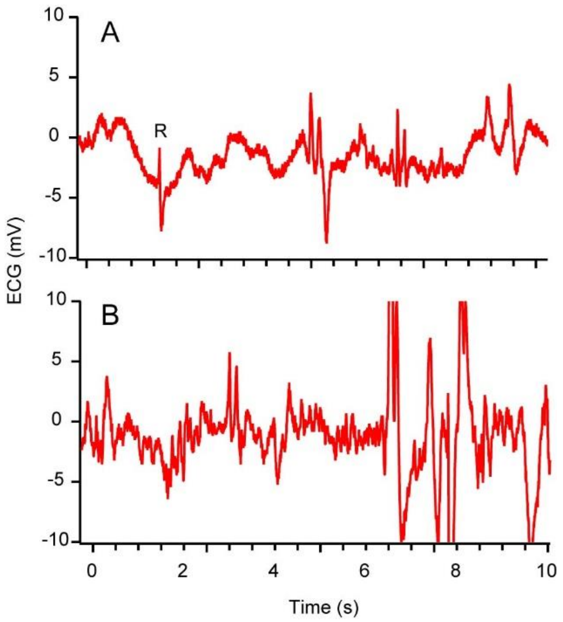

Fig. S3. Typical ECG traces including noise. (A) only one R peak and (B) No R peaks were identified from the ECG trace. We did not use such ECG traces including noise for our analyses.

Biology Open • Supplementary information
